# Extra-Neural Metastases From Primary Intracranial Ependymomas: A Systematic Review

**DOI:** 10.3389/fonc.2022.831016

**Published:** 2022-04-27

**Authors:** Paolo Palmisciano, Gianluca Ferini, Fabio Barone, Vishal Chavda, Fabrizio Romano, Paolo Amico, Donatella Emmanuele, Giovanni F. Nicoletti, Gianluca Pompili, Giuseppe Roberto Giammalva, Rosario Maugeri, Domenico Gerardo Iacopino, Lidia Strigari, Tseng T. Yeo, Salvatore Cicero, Gianluca Scalia, Giuseppe Emmanuele Umana

**Affiliations:** ^1^Department of Neurosurgery, Trauma Center, Gamma Knife Center, Cannizzaro Hospital, Catania, Italy; ^2^Department of Radiation Oncology, REM Radioterapia srl, Viagrande, Italy; ^3^Department of Pathology, Stanford School of Medicine, Stanford University Medical Center, Palo Alto, CA, United States; ^4^Medical Oncology Unit, Umberto I Hospital, Siracusa, Italy; ^5^Department of Pathological Anatomy, Cannizzaro Hospital, Catania, Italy; ^6^Department of Neurosurgery, Highly Specialized Hospital of National Importance “Garibaldi”, Catania, Italy; ^7^Plastic Surgery Unit, Cannizzaro Hospital, Catania, Italy; ^8^Unit of Neurosurgery, Department of Biomedical Neurosciences and Advanced Diagnostics, University of Palermo, Palermo, Italy; ^9^Department of Medical Physics, IRCCS Azienda Ospedaliero-Universitaria di Bologna, Bologna, Italy; ^10^Department of Surgery, Division of Neurosurgery, National University Hospital, Singapore, Singapore

**Keywords:** ependymoma, extra-neural metastases, neuro-oncology, scalp metastases, systematic review

## Abstract

**Background:**

Primary intracranial ependymomas (IE) are rare brain tumors rarely metastasizing outside the central nervous system. We systematically reviewed the literature on extra-neural metastases from primary IEs.

**Methods:**

PubMed, Scopus, Web-of-Science, and Cochrane were searched following the PRISMA guidelines to include studies of extra-neural metastases from primary IEs. Clinical features, management strategies, and survival were analyzed.

**Results:**

We collected 48 patients from 43 studies. Median age was 13 years (range, 2-65). Primary IEs were frequently located in the parietal (22.9%) and frontal (16.7%) lobes, and mostly treated with resection (95.8%) and/or radiotherapy (62.5%). Most IEs were of grade-III (79.1%), and few of grade-I (6.3%) or grade-II (14.6%). 45 patients experienced intracranial recurrences, mostly treated with resection (86.7%), radiotherapy (60%), and/or chemotherapy (24.4%). Median time-interval from primary IEs was 28 months (range, 0-140). Most extra-neural metastases were diagnosed at imaging (37.5%) or autopsy (35.4%). Extra-neural metastases were multifocal in 38 patients (79.1%), mostly involving cervical or hilar lymph-nodes (66.7%), lung/pleura (47.9%), and/or scalp (29.1%). Surgical resection (31.3%), chemotherapy (31.3%) and locoregional radiotherapy (18.8%) were the most common treatments for extra-neural metastases, but 28 (58.3%) patients were not treated. At last follow-up, 37 patients died with median overall-survivals from primary IEs of 36 months (range, 1-239), and from extra-neural metastases of 3 months (range, 0.1-36). Overall-survival was significantly longer in patients with grade-I and II IEs (P=0.040).

**Conclusion:**

Extra-neural metastases from primary IEs are rare, but mostly occur at later disease stages. Multidisciplinary management strategies should be intended mostly for palliation.

## Introduction

Ependymomas are central nervous system (CNS) tumors with a prevalent occurrence in pediatric patients between 0 and 4 years of age (1). The most recent 2021 World Health Organization (WHO) classification of CNS tumors makes a distinction of ependymomas based on a combination of histomolecular features and anatomical locations, dividing them into molecular groups within the supratentorial space, the posterior fossa, and the spinal compartments (2). In adults, approximately 46% of all ependymomas involve the spine, contrarily to ependymomas in children and adolescents that have an overall preferred intracranial location (90%) (3). As regards high-grade tumors, grade-III ependymomas, previously defined as “anaplastic”, identify entities composed of poorly differentiated ependymal cells with intense mitotic activity associated with microvascular proliferation and tumor necrosis (4). Indeed, grade III intracranial ependymomas (IEs) have an high likelihood to recur, and may also spread to distant CNS regions *via* the cerebrospinal fluid, causing the so-called “drop metastases” (3). Extracranial metastases from brain tumors have been reported in approximately 0.5-1% of cases, of which ependymomas comprise the 3.7% in the pediatric population (5). Indeed, IEs rarely metastasize outside the CNS, with only few cases of extra-neural metastases reported in the literature, mostly involving regional lymph nodes, lungs, and scalp (6–8). Extra-CNS metastases of IEs appear to majorly worse patient’s prognosis, owing to the complex planning of multidisciplinary management strategies to contain further spreading of systemic tumor cells (9). In this study, we systematically reviewed the literature on extra-neural metastases from primary IEs, analyzing clinical features, management strategies, and their impact of patients’ prognosis.

## Material and Methods

### Literature Search

A systematic review was completed according to the Preferred Reporting Items for Systematic Reviews and Meta-Analyses (PRISMA) statement and registered to PROSPERO (ID: 289645) (10). PubMed, Scopus, Web of Science, and Cochrane were searched from database inception to 26^th^ October 2021 operating the combination of the Boolean full-text search: [(ependymoma OR ependymal tumor) AND (metastases OR metastasis OR extra-neural)]. Collected studies were exported to Mendeley and duplicates were removed.

### Study Selection

Inclusion and exclusion criteria were set a priori. Studies written in English were included if they involved ≥1 patients with histologically confirmed extra-neural metastases arising from primary IEs. Extra-neural metastases were defined as lesions located outside the CNS in patients with prior pathologically confirmed IEs. Studies were excluded if they reported patients with CNS metastases or skull/scalp lesions deriving from direct invasion from the primary intracranial tumor.

Two independent reviewers (P.P. and G.F.) screened titles and abstracts of all collected citations, and then assessed full-texts of studies that met the inclusion criteria. Disagreements were settled by a third author (G.E.U.). Eligible studies were included based on the pre-determined criteria. References were reviewed to retrieve additional relevant articles.

### Data Extraction

Two independent reviewers (G.S. and P.P.) extracted data from included articles, then confirmed independently by one additional reviewer (G.E.U.). Missing data are either not originally reported or could not be differentiated from other data. The following patient-level data were extracted from all included articles when available: age, gender, primary IE location, primary IE treatment, histopathological WHO grading valid at the time of diagnosis and treatment, presence of intracranial recurrence and relative treatments, time-interval between primary IE and onset of extra-neural metastases, extra-neural metastases’ location and eventual treatment, overall survival (OS) from diagnoses of primary IE and extra-neural metastases, survival status.

### Data Synthesis and Quality Assessment

The primary outcomes of interest were clinical characteristics and management strategies of patients with extra-neural metastases from primary IE. For each article, two authors (P.P. and G.S.) independently appraised level of evidence upon the 2011 Oxford Centre For Evidence-Based Medicine guidelines, and also assessed risk of bias using the Joanna Briggs Institute checklists for case-reports and case-series (11–13). Meta-analysis was precluded because all included articles had levels IV-V of evidence, and hazard ratios could not be deducted.

### Statistical Analysis

The software SPSS V.25 (IBM Corp, Armonk, New York) was used to run all analyses. Continuous variables are reported as means or medians and ranges, while categorical variables are presented as frequencies and percentages. OS curves from primary IE’s diagnosis and extra-neural metastases’ occurrence were estimated using the Kaplan-Meier method and the survival analyses obtained with the log-rank test. A two-tailed P-value <0.05 was considered statistically significant for all analyses.

## Results

### Study Selection

**Figure 1:**
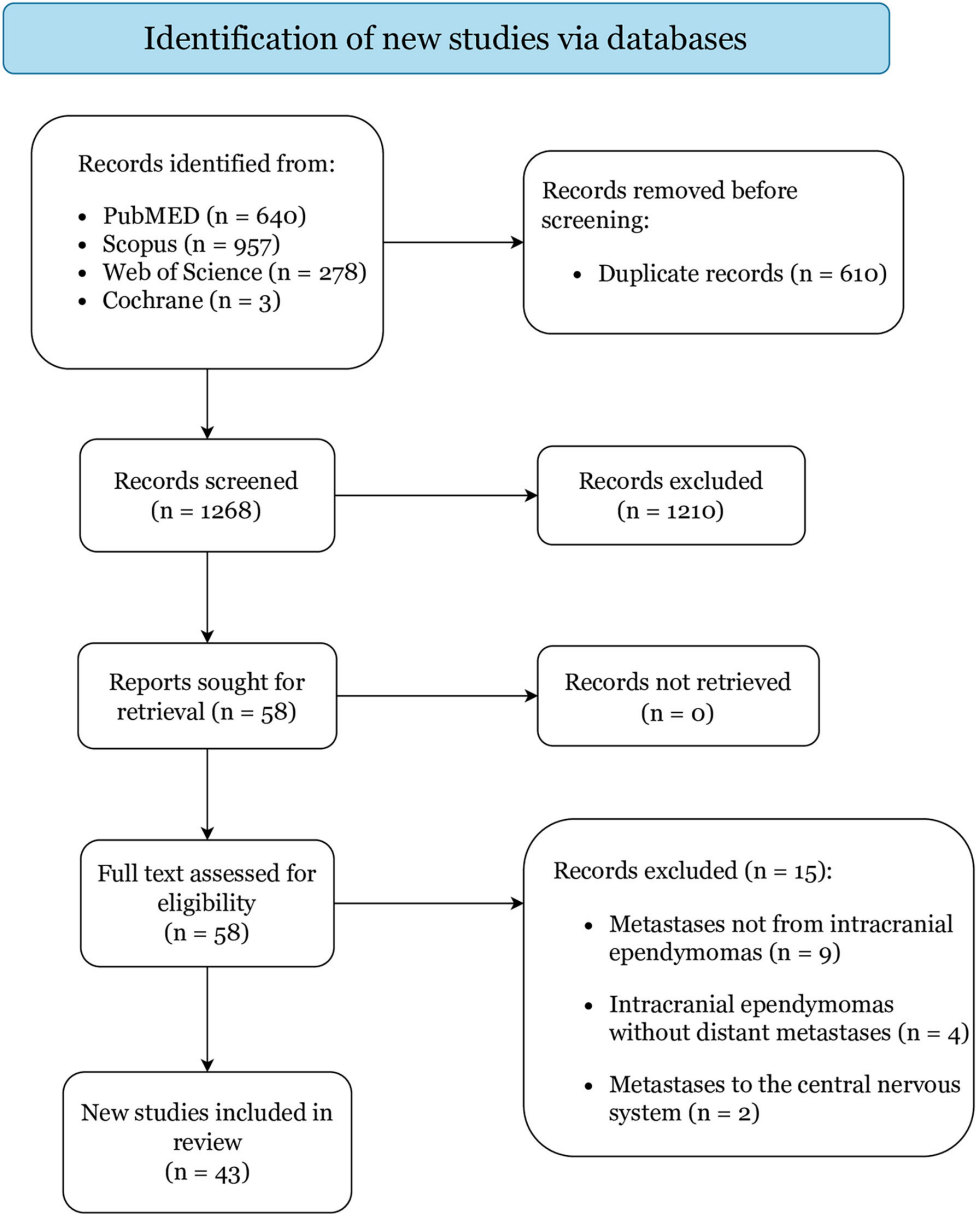
PRISMA 2020 flow diagram.

**Figure 1** displays the literature search and study selection process. In total, 43 articles reporting on 48 cases were included: 5 were case-series (including 10 patients) and 38 were case-reports, categorized as level IV and V of evidence, respectively (**Table 1**) (6–9, 14–52). Quality assessment resulted in low risk of bias for all included studies (**Supplementary File 1**).

**Table 1:** Overview of all included studies.

	Authors, Year	Study Design Level of evidence	Age, Gender	Location Primary Tumor	WHO Grade	Treatment Primary Tumor	Intracranial Recurrences	Treatment Recurrences	Time between primary & extra-neural metastases (months)	Extra-neural Metastases Location	Diagnoses Extra-neural Metastases		Treatment Extra-neural Metastase	Survival from diagnosis of primary (months)	Survival from diagnosis of metastases (months)	Status
1	**Maass, 1954** (14)	Case report – V	27, M	Occipital	IV	Resection	Yes	S (2) + RT	28	CLN, Sc	Biopsy	S		30	2	Dead
2	**Sherbaniuk and** Shnitka**, 1956** (15)	Case report – V	16, F	Parietal	I	Resection	Yes	S (2) + RT	140	L/P, Sc	Clinical		No	156	14	Dead
3	**Breslich, 1957** (16)	Case report – V	13, M	Occipital	III	Resection	Yes	S (1) + RT	Autopsy	HLN, L/P	Autopsy		No	24	0	Dead
4	**Perry, 1957** (49)	Case report – V	39, M	Parietal	I	Resection + RT	Yes	N/A	Autopsy	L, LB	Autopsy		No	72	0	Dead
5	**Wen and Barrows, 1957 (** 17**)**	Case report – V	9, M	Frontal	I	Resection	Yes	S (1) + RT	Autopsy	HLN, L/P, Sc	Autopsy		No	14	0	Dead
6	**Perrin, 1958** (18)	Case series – IV	2, F	Cerebellum	IV	Resection + RT	Yes	S (1)	Autopsy	HLN, L/P	Autopsy		No	7	0	Dead
			4, F	Thalamus	IV	Biopsy + EVD + RT	No	N/A	Autopsy	HLN, LB, V	Autopsy		No	14	0	Dead
7	**Nigogosyan, 1962** (19)	Case report – V	15, F	Parietal	II	Resection	Yes	S (2) + CT + RT	Autopsy	L/P	Autopsy		No	54	0	Dead
8	**Glasauer and Yuan, 1963** (20)	Case report – V	2, M	Parieto-occipital	IV	Resection + RT	Yes	S (1)	13	HLN, L/P	Imaging		No	14		Dead
9	**MacMahon and Urista, 1964** (21)	Case report – V	3, M	Lateral ventricle	IV	Resection + RT	Yes	S (1)	Autopsy	HLN, L/P	Autopsy		No	15	0	Dead
10	**Hesselvik and** Tygstrup**, 1965** (22)	Case report – V	2, M	Parietal	IV	Resection + RT	Yes	S (2)	Autopsy	L/P, Sc	Autopsy		No	12	0	Dead
11	**Fragoyannis and Yalcin, 1966** (23)	Case series – IV	22, M	Occipital	III	Resection	Yes	S (1)	9	CLN, V	Biopsy		No	11	2	Dead
			7, F	Fourth ventricle	III	Resection	No	N/A	10	LB, Sc	Imaging		S + RT	16	6	Dead
12	**Wentworth and Birdsell, 1966** (18)	Case report – V	2, M	Parieto-occipital	IV	Resection + RT	Yes	S (1)	Autopsy	HLN, L/P	Autopsy		No	6	0	Dead
13	**Robinson and Sharkey, 1967** (24)	Case report – V	14, M	Third ventricle	IV	Resection + RT	Yes	S (1)	Autopsy	HLN, L/P	Autopsy		No	20	0.1	Dead
14	**Braun and Tzonos, 1968** (25)	Case report – V	6, F	Frontal	II	Resection + RT	Yes	S (1)	2	Sc	Clinical		No	5	0	Dead
15	**Smith et al., 1969** (26)	Case report – V	31, M	Parietal	II	Resection	Yes	S (1) + RT	Autopsy	CLN, L/P	Autopsy		No	96	0	Dead
16	**Hojgaard and Johansen, 1970** (27)	Case report – V	13, F	Fourth ventricle	IV	Resection + RT	Yes	S (1)	Autopsy	K, L, L/P	Autopsy		No	9	0	Dead
17	**Schuster et al., 1976** (28)	Case series – IV	13, F	Fronto-parietal	III	Resection	Yes	S (1)	7	CLN	Biopsy		No	8	1	Alive
			7, M	Parietal	III	Resection + RT	Yes	S (1) + RT	28	CLN, Sc	Clinical		No	31	3	Dead
18	**Tamura et al., 1980** (29)	Case report – V	22, F	Frontal	IV	Resection + RT	Yes	S (1)	Autopsy	L, L/P	Autopsy		No	22	0	Dead
19	**Duffner and Cohen, 1981** (30)	Case report – V	9, F	Parietal	II	Resection + RT	Yes	S (2) + CT	Autopsy	HLN, LB, L/P	Autopsy		No	45	0	Dead
20	**Panyathanya and** Chantarakul**, 1982** (31)	Case report – V	38, F	Lateral ventricle	II	Resection	No	N/A	Autopsy	Heart	Autopsy		No	1	0	Dead
21	**Andoh et al., 1984** (32)	Case report – V	28, F	Parietal	III	Resection	Yes	S (2) + RT	38	HLN, L/P, Sc, V	Imaging		RT	63	25	Dead
22	**Ferracini et al., 1984** (33)	Case report – V	30, F	Temporal	IV	Resection + RT	Yes	RT	10	CLN	Biopsy		S + RT	17	6	Dead
23	**Wakabayashi et al., 1986 (** 34**)**	Case report – V	19, M	Frontal	IV	Resection + RT	Yes	S (1) + RT	Autopsy	C/HLN, LB, L/P	Autopsy		No	29	0	Dead
24	**Lioté et al., 1988 (** 35**)**	Case report – V	27, F	Occipital	IV	Resection + RT	Yes	S (5) + RT	24	L/P	Imaging		S + CT	168	18	Dead
25	**Itoh et al., 1990** (36)	Case report – V	59, M	Fourth ventricle	IV	Resection + RT	Yes	S (1) + RT	26	L/P, Skin	Imaging+biopsy		S + CT + RT	36	9	Dead
26	**Newton et al, 1992** (9)	Case series – IV	3, M	Thalamus/Third ventricle	III	Biopsy+EVD+CT+RT	Yes	CT + RT	27	HLN, P	Clinical+imaging		S + CT	36	9	Alive
			3, M	Fronto-parietal	III	Resection + RT	Yes	S (1) + CT	Autopsy	HLN, L/P	Autopsy		No	18	0	Dead
27	**Fouladi et al., 2003** (37)	Case series – IV	14, F	Frontal	II	Resection	Yes	S (1)	N/A	CLN	Clinical		S + CT + RT	69	N/A	Dead
			8, M	Occipital	II	Resection	Yes	S (2)	N/A	CLN, ST	Clinical		S + CT + RT	239	N/A	Dead
28	**Strunk et al., 2004 (** 38**)**	Case report – V	15, M	Temporo-parietal	III	Resection + CT	Yes	S (2) + CT + RT	84	HLN, L/P	Clinical+imaging		No	87	3	Dead
29	**Kinoshita et al., 2004** (39)	Case report – V	11, F	Frontal	III	Resection + RT	Yes	S (8) + RT	36	CLN	Clinical		S + CT	72	36	Alive
30	**Kumar et al., 2007** (40)	Case report – V	10, M	Lateral ventricle	III	Resection	Yes	S (2) + CT + RT	108	CLN, Sc	Biopsy		S + CT	120	12	Alive
31	**Donepudi et al., 2009** (41)	Case report – V	57, M	Parietal	III	Resection + CT + RT	Yes	S (4) + CT + RT	84	CLN, Sc	Biopsy		S + CT	108	7	Alive
32	**Hussain et al., 2010** (42)	Case report – V	6, F	Frontal	III	Resection + RT	Yes	S (4) + RT	68	LB	Imaging		CT	72	4	Dead
33	**Chao et al., 2011** (43)	Case report – V	10, M	Parietal	III	Resection	Yes	CT + RT	18	CLN, Sc	Imaging		CT	43	25	Dead
34	**Davis et al., 2011** (6)	Case report – V	22, F	Fronto-temporal	III	Resection + RT	Yes	RT	12	CLN, LB, PG, Sc	Biopsy		S + RT	54	36	Alive
35	**Fischer et al., 2013** (40)	Case report – V	6, F	Parietal	III	Resection + RT	Yes	S (1) + CT	27	V	Imaging		CT + RT	36	9	Dead
36	**Alzahrani et al., 2014** (8)	Case report – V	7, M	Occipital	III	Resection+VPS+CT+RT	Yes	S (1) + RT	60	L/P	Imaging		No	72	12	Alive
37	**Pachella et al., 2015** (7)	Case report – V	21, M	Temporo-parietal	III	Resection	Yes	S (3) + CT + RT	58	CLN	Imaging+biopsy		CT	72	14	Alive
38	**Tyzo et al., 2015** (44)	Case report – V	22, M	Parieto-occipital	III	Resection + RT	Yes	S (1) + CT + RT	55	CLN, Sc	Imaging+biopsy		No	58	3	Alive
39	**Kim et al., 2017** (45)	Case report – V	10, M	Fronto-parietal	III	Resection + RT	Yes	S (2) + RT	84	HLN,L,LB,L/P,Sc	Imaging		S + CT	108	24	Dead
40	**Marsecano et al., 2017** (46)	Case report – V	65, F	Temporo-parietal	III	Resection	Yes	RT	7	HLN, L, V	Imaging		No	7	0.1	Dead
41	**Umbach et al., 2019** (47)	Case report – V	17, F	Frontal	III	Resection + RT	Yes	RT	72	CLN, PG	Imaging+biopsy		S + CT + RT	84	14	Alive
42	**Shunnan et al., 2020** (48)	Case report – V	2, F	Parieto-occipital	III	Resection + VPS + RT	Yes	S (2)	21	L/P	Imaging		No	25	4	Dead
43	**St Jeor et al., 2020** (49)	Case report – V	1, M	Occipital	III	Resection + VPS + RT	Yes	S (3) + RT	48	P, V	Imaging		S + CT	60	12	Alive

CLN, Cervical lymph nodes; CT, Chemotherapy; EVD, External ventricular drainage; , Female; HLN, Hilar lymph nodes; K, Kidney; L, Liver; LB, Long bones; L/P, Lung/Pleura; M, Male; N/A, Not Available; P, Peritoneum; PG, Parotid gland; RT, Radiotherapy; S, Surgery; Sc, Scalp; ST, Soft tissue; V, Vertebrae; VPS, Ventriculoperitoneal Shunt.

### Demographics and Primary Tumor Characteristics

Patients aged from 2 to 65 years with a median of 13 years and were predominantly males (56.3%) (**Table 2**). Primary IEs were mostly supratentorial (44, 91.7%) and rarely infratentorial (4, 8.3%), most frequently located in the parietal (22.9%), frontal (16.7%), and occipital (14.6%) lobes. Surgical resection was completed in most cases (95.8%), and only 2 pediatric patients (4.2%) with inoperable thalamic tumors received biopsy only (9, 18). Adjuvant radiotherapy was administered in 30 cases (62.5%) and adjuvant chemotherapy in 4 (8.3%). CSF shunting was also performed in 5 patients (10.4%) with severe hydrocephalus and consequent intracranial hypertension (8, 9, 18, 48, 49). At histological examination, most tumors were of grade-III (79.1%), and only few neoplasms were of low-grade (grade-I 6.3%; grade-II 14.6%). A total of 45 patients (93.8%) experienced one or multiple IE recurrences, with a median of 2 IE recurrences per-patient (range, 1-8). Most IE recurrences were treated with surgical resection (86.7%), often coupled with adjuvant brain radiotherapy (60%), including external beam radiotherapy (44.4%) and gamma knife radiosurgery (15.6%), and/or adjuvant systemic chemotherapy (24.4%).

**Table 2:** Summary of clinical characteristics and management strategies of all pooled primary intracranial ependymomas.

Characteristics	Value
Cohort size (no.)	48
Demographics	
Median age, range (years)	13, 2–65
Gender (male)	27 (56.3%)
Location Primary Intracranial Ependymomas	No. (%)
Parietal	11 (22.9%)
Frontal	8 (16.7%)
Occipital	7 (14.6%)
Parieto-occipital	4 (8.3%)
Fourth ventricle	3 (6.3%)
Fronto-parietal	3 (6.3%)
Lateral ventricle	3 (6.3%)
Parieto-temporal	3 (6.3%)
Thalamus/Third ventricle	3 (6.3%)
Cerebellum	1 (2.1%)
Fronto-temporal	1 (2.1%)
Temporal	1 (2.1%)
Treatment Primary Intracranial Ependymomas	No. (%)
Surgery	48 (100%)
Resection	46 (95.8%)
Biopsy	2 (4.2%)
Radiotherapy	30 (62.5%)
Chemotherapy	4 (8.3%)
External/Vetriculoperitoneal Shunt	5 (10.4%)
Histological WHO Grade	No. (%)
Grade I	3 (6.3%)
Grade II	7 (14.6%)
Grade III – “Anaplastic Ependymoma”	38 (79.1%)
Intracranial Recurrence	45 (93.8%)
Per-patient number of recurrences, median (range)	2 (1 – 8)
Treatment of Intracranial Recurrences (n = 45)	No. (%)
Surgical resection	39 (86.7%)
Per-patient number of surgeries, median (range)	2 (1 – 8)
Radiotherapy	27 (60%)
External Beam radiotherapy	20 (74.1%)
Gamma Knife	7 (25.9%)
Chemotherapy	11 (24.4%)

### Clinical Features, Management, and Outcomes of Extra-Neural Metastases

**Table 3** summarizes clinical features and treatment outcomes of all pooled extra-neural metastases. Median time-interval between primary IE diagnosis and extra-neural metastases occurrence was 28 months (range, 2-140). Most extra-neural metastases were diagnosed at imaging (37.5%), post-biopsy histopathology (22.9%), and/or clinical examination (16.7%), but 17 metastases (35.4%) were diagnosed only at post-mortem autopsy. In most cases (79.1%), ependymomas metastasized in multiple extra-neural locations, most frequently involving the cervical or hilar/mediastinal lymph nodes (66.7%), the lungs and pleura (47.9%), and the scalp (29.1%). Of note, Panyathanya et al. (31) reported an unusual case of a cardiac ependymoma metastases diagnosed at autopsy in one patient with lateral ventricle IE who suddenly died 1 month after primary tumor resection.

**Table 3:** Summary of clinical characteristics, management strategies, and survival of all pooled extra-neural metastases.

Characteristics	Value
Diagnosis Extra-neural Metastases	No. (%)
Time interval from primary, median (range) (months) (not autopsy)	28 (2 – 140)
Imaging	18 (37.5%)
Autopsy	17 (35.4%)
Biopsy/Pathology	11 (22.9%)
Clinical exam	8(16.7%)
Location Extra-neural Metastases	No. (%)
Multiple locations	38 (79.1%)
Lymph nodes	32 (66.7%)
Cervical	17 (35.4%)
Mediastinal/Hilar	17 (35.4%)
Lung/Pleura	23 (47.9%)
Scalp	14 (29.1%)
Bones	13 (27.1%)
Long bones	8(16.7%)
Vertebrae	6(12.5%)
Liver	4(8.3%)
Mediastinum	2 (4.2%)
Parotid gland	2 (4.2%)
Peritoneum	2 (4.2%)
Heart	1 (2.1%)
Kidney	1 (2.1%)
Skin	1 (2.1%)
Soft tissue	1 (2.1%)
Treatment Extra-neural Metastases	No. (%)
Surgery	15 (31.3%)
Chemotherapy	15 (31.3%)
Radiotherapy	9(18.8%)
No treatment	28 (58.3%)
Overall Survival (months)	Median (range)
From primary tumor diagnosis	36 (1 – 239)
From extra-neural metastases diagnosis (not autopsy)	3 (0.1 – 36)
Status	No. (%)
Alive	11 (22.9%)
Dead	37 (77.1%)

**Figure 2:**
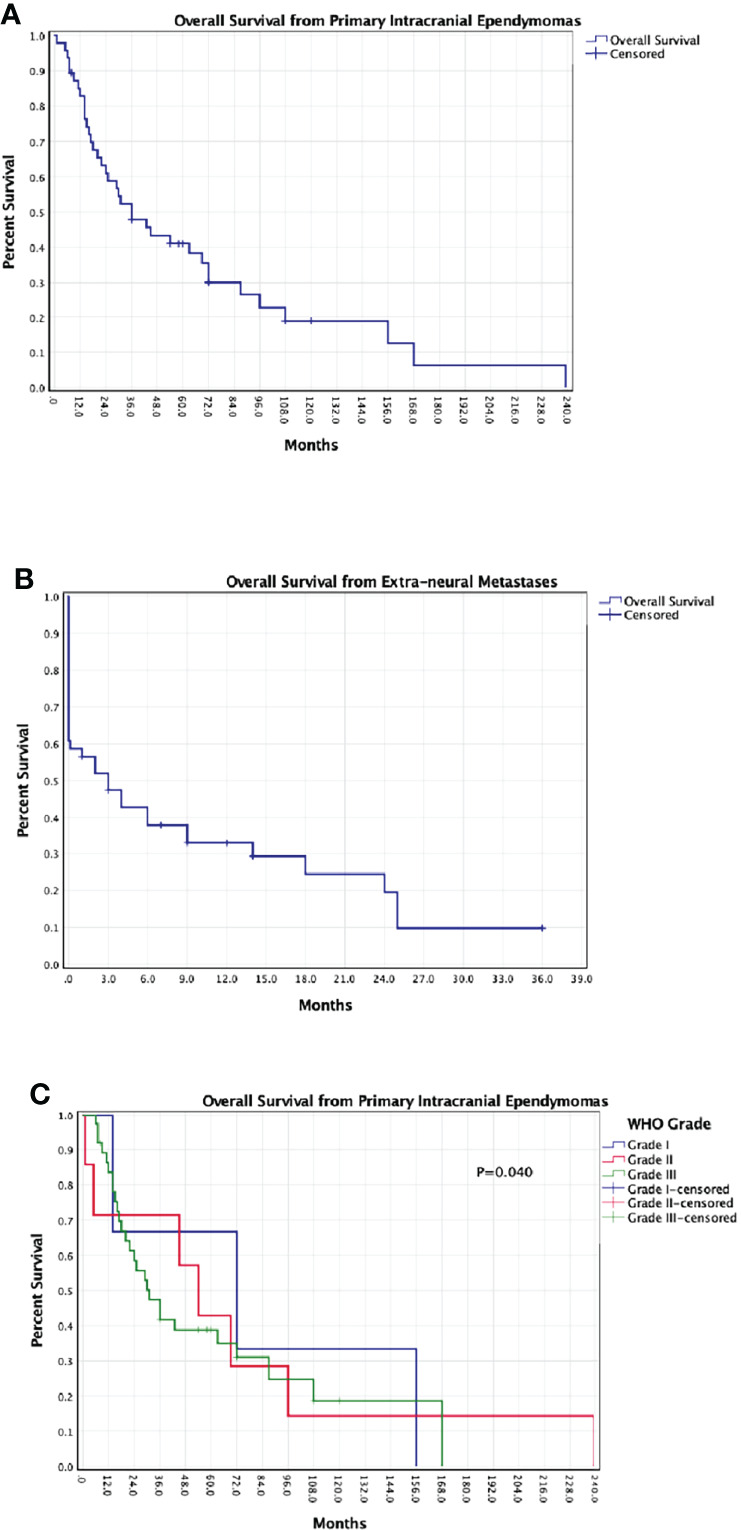
Kaplan-Meier survival curves of all pooled patients: **(A)** overall survival from diagnosis of primary intracranial ependymoma; **(B)** overall survival from diagnosis of extra-neural metastases; **(C)** overall survival from diagnosis of primary intracranial ependymoma based on WHO grade.

Extra-neural metastases were treated in 20 patients (41.7%) with surgical resection (31.3%), systemic-chemotherapy (31.3%), and/or locoregional radiotherapy (18.8%). However, a total of 28 patients (58.3%) did not receive any treatment for their extra-neural metastases. At last available follow-up, 37 patients (77.1%) died with a median OS from primary IE diagnosis of 36 months (range, 1-239), and a median OS from extra-neural metastases occurrence of 3 months (range, 0.1-36) (**Figure 2**). OS was significantly longer in patients with low-grade (grade-I and grade-II) ependymomas (median 61.5 months; range, 1-239) as compared to patients with grade-III ependymomas (median 30.5 months; range, 6-168) (P=0.040).

## Discussion

This systematic review focuses on extra-neural metastases from primary IEs. A previous review on extra-neural metastases included only patients with “anaplastic” grade-III IE, but did not include 32 of the present studies, most likely due to changes in WHO criteria for CNS ependymomas (47).

### Intracranial Ependymomas’ Spreading Routes

While CSF-borne leptomeningeal dissemination represents a more frequent yet challenging complication of CNS tumors, extra-neural metastases are rare and often difficult to spot (53, 54). Extracranial metastases from brain tumors have been reported in approximately 0.5-1% of cases, of which ependymomas comprise the 3.7% in the pediatric population (5). More recently, the increased incidence of such entities possibly reflects the improved management and superior survival observed in the current neuro-oncology practice (55, 56). Hence, a better understanding of CNS and extra-CNS metastasizing pathways may serve an important role for the multidisciplinary treatment of patients with brain tumors (57, 58). Extra-neural metastases of CNS tumors have been hypothesized to develop from several different routes. While hematogenous spread of neoplastic cells may be caused by pathologic vessels sprouting within the tumor or infiltration of dural sinuses, lymphatic spread may follow tumor’s invasion of the skull, scalp, and related extracranial soft tissues, followed by embolization of clusters of neoplastic cells (59–61). Although these pathways were mainly described for other pediatric and adult CNS tumors, including medulloblastomas and glioblastomas, such routes were also deemed responsible for most extra-neural metastases from IEs (57, 62, 63). This is further supported by the fact that the most frequent secondary lesions found in our review described the involvement of lymph nodes, lungs, and scalp (7, 8, 43, 48). The presence of multifocal extra-neural metastases in most of our pooled patients also suggests that hematogenous and lymphatic routes are not mutually exclusive but may coexist, especially in aggressive grade-III IEs (6, 30, 32, 34). Of note, the singular cases of parotid glands (43, 47), renal (27), and cardiac (31) IE metastases likely imply the influence of specific “host organs” histomolecular and immune microenvironments in regulating tumor growth and metastatic patterns (64–68). From here, the importance of developing targeted immunotherapies extends beyond the most common gliomas and brain metastases to the less frequent and rare CNS tumors, including IEs, which have a major impact in routine neuro-oncology patient care (69, 70). Of interest, we noted that extra-CNS IE metastases were diagnosed only based on patient’s history and clinical presentation, without histological confirmation, in 37.5% of patients due to ineligibility or refusal to undergo surgery. In these complex cases, the authors confirmed the importance of providing prompt diagnosis and management to allow improvement of patient’s quality of life and prevent further worsening of their prognoses.

### Impact of Surgery in Intracranial Ependymomas’ Extra-Neural Spreading

Surgical resection and manipulation have been suggested to play a role in extra-neural metastasizing of primary CNS tumors, but the evidences are somewhat contradictory (57, 71, 72). Some authors theorized that the osteo-meningeal gaps created by the operation, in combination with extra-meningeal tissues’ exposure to tumor cells and their direct intraoperative transfer *via* surgical instruments, likely allows malignant cells to access the blood and lymphatic vessels and embolize to distant organs (6, 57, 71). Further facilitating mechanisms include the formation of fragile vessels during the postoperative re-capillarization period, which may be easily invaded by neoplastic cells, and/or the use of adjuvant brain radiotherapy protocols, which may loosen up dura’s cellular junctions favoring trans-dural migration of tumor cells (73, 74). In support of these hypotheses, all pooled patients in our review received surgery for their primary IEs, often coupled with adjuvant radiotherapy protocols. In addition, the vast prevalence of multiple intracranial IEs recurrences (94%) and repetitive surgical manipulations (87%) may have contributed to tumor cells embolization and extra-neural spreading (43, 47). However, other authors cast doubts of the sole role of surgery in extra-neural spreading of CNS tumors, observing the occurrence of extra-neural metastases in non-surgical gliomas or the presence of circulating glioma-cells before performing surgical tumor resection (72, 75). In our cohort, some large extra-neural metastases have been detected only few months after the completion of single neurosurgical procedures for primary IEs, likely supporting the fact that surgery alone is not sufficient to explain extra-CNS spreading patterns (25, 28, 46). Of note, Newton et al. (9) and St Jeor et al. (49) also hypothesized the likely peritoneal spreading of tumor cells *via* ventriculoperitoneal shunts, in line with previous reports for other CNS tumors; still the evidences are limited, and further research is needed (76, 77).

### Patterns Showing a More Aggressive Course

The relationship between IE’s WHO grade and their likelihood to metastasize is still poorly understood. In 2019, Umbach et al. (47) reviewed the literature on extra-neural metastases only from grade-3 IEs, previously called “anaplastic”, suggesting a positive but unclear correlation with their aggressive nature. In our review, we similarly found that most of patients with extra-neural IE metastases were diagnosed with high-grade tumors (79.6%), but the presence of 10 patients (20.4%) with low-grade IEs needs to be mentioned. We presume that the difficulty to clearly identify the link between histopathological grading and extra-CNS spreading likely stems from the frequent changes in WHO grading criteria, which may have been responsible for an underestimation of the high-grade IE cases. This may also explain the similar Kaplan-Meier survival curves noted for grade-II and grade-III IEs, confirming a worse prognosis compared to grade-I lesions but failing to show a significant differences between the two. The advances introduced in the 2021 WHO classification (2), which includes also a detailed histomolecular tumor analysis are expected to provide better prognostic and survival data within future studies on primary IEs and extra-CNS metastases. By analyzing our pooled patients in line with the recently updated 2021 WHO classification (2), we noted that most extra-neural metastases originated from supratentorial IEs (91.8%) and rarely from infratentorial IEs (8.2%), further highlighting the importance to differentiate these two entities. Our findings may lead to the conclusion that supratentorial ependymomas show a higher tendency to spread outside the CNS as compared to infratentorial ependymomas. However, we emphasize that the rarity of extra-neural IEs metastases preclude a definite explanation, as the different management strategies adopted for the two anatomical IEs’ variants may have also played a role. Indeed, similarly to the current data on brain metastases and gliomas (78, 79), surgical resection may be preferred for supratentorial tumors due to the favorable surgical access and tumor exposure, while less invasive and non-operative strategies, including tumor biopsy and chemo/radiotherapy for infratentorial IEs, with the goal of reducing the risks of surgical-related complications. As surgery may be related to extra-neural metastases mechanisms, the actual impact of IEs’ grading based on anatomical location remains unclear.

### Histomolecular Characteristics

The recent molecular characterization and classification of IEs led to major implications in better understanding such tumors and tailoring appropriate management strategies. Ten subgroups, each carrying singular molecular features, have been identified and divided based on anatomical location: three supratentorial, three infratentorial, three spinal, and one (subependymoma) found in all three compartments (2). Supratentorial ependymomas encompass: 1) supratentorial ependymomas with YAP1-fusion, grade 2-3 pediatric tumors with good prognosis, which maybe targeted by YAP/TAZ inhibitors such as dasatinib and pazopanib; 2) supratentorial ependymomas with ZFTA-fusion, grade 3 entities with worse prognosis and intraventricular predilection, previously classified as RELA-fusion positive; 3) supratentorial ependymomas NOS/NEC, grade 2-3 tumors with no-YAP1/ZFTA fusion, which represent a subgroup of exclusion (80). Infratentorial ependymomas include: 1) posterior fossa ependymomas group A, grade 3 tumors with poor prognosis, loss of H3K27me3 expression, and EZHIP mutations, whose pharmaceutical interruption may block tumorigenesis; 2) posterior fossa ependymomas group B, grade 2 H3K27me3-positive neoplasms with better prognosis and mostly occurring in young adults, characterized by higher chromosomal instability and FOXJ1 hyperactivity; 3) posterior fossa ependymomas NOS/NEC, grade 2-3 lesions with other or not analyzable molecular features that mostly arise from the ependymal lining of the fourth ventricle (80). Spinal ependymomas comprise: 1) myxopapillary ependymomas, grade 2 tumors involving the cauda equina/filum terminale and showing overexpression of HOX, NEFL, and PDGFRA genes; 2) spinal ependymomas, grade 3 lesions characterized by NF2 (Merlin gene) alterations that can be targeted by MEK inhibitors and VEGF inhibitors; 3) spinal ependymomas with MYCN amplification, grade 3 entities with worse prognoses and higher propensity of leptomeningeal spread, but with no available clinical trials on MYCN-targeting inhibitors (80). Lastly, subependymomas are considered slow-growing WHO grade 1 tumors, presenting with CSF obstruction if intracranial or myelopathy/radiculopathy if spinal, and with tumorigenesis likely driven by loss of chromosome 6q and alterations in topoisomerase and p-STAT3/HIF-1α inhibitors, which represent viable molecular therapeutic targets (81). Amongst our included studies, only few data on histomolecular features of extra-neural IE metastases were available, and only one pediatric patient with a RELA-fusion positive (now ZFTA-fusion positive) fronto-parietal IE was confirmed (45). Interestingly, although RELA/ZFTA-fusion positivity has been correlated with poor prognosis in supratentorial IEs, our reviewed case presented a long survival of 10-years, in spite of the presence multifocal systemic metastases (81, 82). Hence, the complexity of IEs’ histomolecular and anatomical grading, their influence on extra-CNS spreading patterns, and the role of potential molecular targets for newer systemic therapies also addressing extra-neural metastases require further analysis.

### Hypotheses on the Most Common Spreading Mechanisms

Scalp and cervical lymph nodes metastases represent two of the most common secondary extra-neural lesions from primary IEs. Itoh et al. (36) documented a relative high incidence of scalp metastasis, suggesting a possible favorable skin environment for the diffusion of ependymoma cells. Similarly, Umbach et al. (47) evidenced the propensity of ependymoma cells to spread through lymphatic pathways towards cervical and/or mediastinal lymph nodes. In some our reviewed pertinent cases, we hypothesize that ependymoma’s extra-neural dissemination may have derived from the intraoperative relocation of tumor-cells to the scalp tissues and their lymphatic spread with cervical lymph nodes’ filtering (39, 44). In addition, the delayed occurrence of such extra-neural metastases, diagnosed years after the initial IE presentation, may be explained by the previously described “cancer cell dormancy” mechanism (83, 84). Of note, although tumor-cell spread through subarachnoid CSF spaces into arachnid villi, superior sagittal sinus, and venous circulation has been widely observed amongst our included studies, no cased of CSF drop metastases were described in our pooled patients, further supporting the theory of lymphatic-borne extra-CNS dissemination.

### Multidisciplinary Management

The multidisciplinary management of extra-neural IE metastases remains complex, and prognoses often unfavorable. Davis et al. (6) reported the occurrence of scalp and cervical nodes ependymoma metastases 1-year after the initial surgery, which evolved into rapid multi-organ diffusion and death 3-year later. We noted that most patients with scalp metastases were managed with seriated resection and external beam radiotherapy, without concurrent systemic chemotherapy, supported by the common agreement that CNS tumors frequently show poor response to chemotherapeutic agents due to their low-rate of blood barrier penetration. Kim et al. (45) also presented a patient with scalp and hilar nodes ependymoma metastases occurring 7-years after neurosurgery, treated with etoposide, ifosfamide and cisplatin, but showing poor response with progressive worsening and death 2-years later. In contrast, Kumar et al. (40) reported a good response to etoposide with total regression of the scalp and cervical nodes ependymoma metastases, suggesting an effective clinical outcome associated to this chemotherapeutic regimen. Hence, in consideration of the extra-cranial location of scalp and cervical lymph nodes metastasis, the response to adjuvant chemotherapy and radiotherapy protocols should be expected to be more favorable, and strongly considered in the management of these patients. Finally, neuroplastic surgical procedures play an important role in cases of giant and disfiguring scalp metastases, achieving optimal therapeutic and esthetic outcomes with high patient satisfaction.

### Palliative Radiotherapy

Reports on prescribed doses to the extracranial ependymoma metastases are absent in many of our included studies (28, 30). There is no specific treatment paradigm as regards the management of extracranial ependymoma metastases from the radiation oncologist’s point of view. In some case reports, when not delivered as first-line palliative treatment, adjuvant radiotherapy has been administered to the metastatic site after maximal surgical resection. Due to the very poor prognosis of these patients, classic palliative radiation schemes may represent an adequate option to achieve favorable symptom relief, including 30 Gy in 10 daily fractions, 20 Gy in four daily fraction, and 8 Gy in single fraction. The main issue with such palliative doses is that they may lead to poor local tumor control as reported by Fisher et al. (51) in a child with T1 bony secondary lesion irradiated with 30 Gy in 10 fractions. However, while higher radiation doses may not provide better results, as reported by Davis et al. (6) who irradiated ependymoma skin metastases with a total dose of 54 Gy, lower doses, such as 8 Gy in single fraction, may result in the metastasis disappearance when integrated with chemotherapy (40). Since most extracranial recurrences develop on the scalp, probably due to surgical seeding, one might debate about the usefulness of prophylactic irradiation of surgical incision sites, as used in the treatment of mesothelioma (43, 85). Ultimately, the paucity and heterogeneity of data do not allow for clear radiation dose recommendations for this patient population. Yet, we note that electron beam radiotherapy, by virtue of a low penetration of radiation, may be preferable and more favorable than photon beam radiotherapy for the treatment of cutaneous metastases (86).

### Limitations

Our study has some limitations, primarily due to the paucity of extra-neural IE metastases in the literature. All the studies included in this review are case-reports or case-series, likely affected by availability and misclassification biases due to the paucity of data and disagreement of WHO grading through the years, respectively. Included studies also covered a 66-year time-period characterized by important advances in neurosurgical and oncological treatments, which likely introduced confounding variables into our analysis. As data was collected retrospectively, this study is limited by the data heterogeneity. The small sample size and low levels of evidence of included articles are prohibitive of performing a meta-analysis. The availability of imaging technology is a limiting factor as some of the included studies were published before MRI or CT were in common usage, thus reporting the detection of extra-neural metastases at autopsy only. Similarly, the inclusion of cases with non-histologically proven extra-CNS metastases may have introduced some bias into our analysis; yet the inclusion only of cases with biopsy-proven primary IEs and clinico-radiologically confirmed extra-CNS metastases likely limited the rates of misdiagnosis and reflected the routine clinical approach used for these complex cases. Finally, due to the frequently missing data on neuropathological and histomolecular features of extra-neural IE metastases across our included studies, we cannot comprehensively analyze the relationship between molecular patterns and IEs’ tendency to metastasize outside the CNS, nor evaluate potential molecular targets for the management of such complex cases.

## Conclusion

Extra-neural metastases from IEs are rare, and mostly occur at later disease stages. The possible mechanisms of extra-CNS tumor-cells dissemination have been analyzed, possibly iatrogenic after surgical resection and/or hematogenous/lymphatic/CSF-borne favored by “host organs” specific microenvironments. The role of histopathologic and anatomical IE grade has been evaluated in the context of the updated 2021 WHO classification of CNS tumors, but the scarce data on histomolecular patterns require further analysis. Multidisciplinary management strategies, including neurosurgical or neuroplastic procedures along with chemoradiotherapy protocols, are highly recommended for scalp and lymph nodes metastases. In view of the poor prognoses of these complex patients, currently treatments should be mostly intended for palliation, but future research needs to be focus on better understanding CNS and extra-CNS tumor microenvironments in order to develop targeted and effective systemic therapies.

## Data Availability Statement

The original contributions presented in the study are included in the article/**Supplementary Material**. Further inquiries can be directed to the corresponding author.

## Author Contributions

Conceptualization, GS and GU. Methodology, PP. Validation, PP, GF, GS, and GU. Formal analysis, PP. Resources, GF, FB, GP, FR, PA, DE, GN, GG, RM, DI, LS, TY, SC, and VC. Data curation, PP. Writing—original draft preparation, PP. Writing—review and editing, GF, FB, GP, FR, PA, DE, GN, GG, RM, DI, LS, TY, SC, GS, GU, and VC. Visualization, PP. Supervision, GS and GU. Project administration, PP, GS, and GU. All authors have read and agreed to the published version of the manuscript.

## Conflict of Interest

The authors declare that the research was conducted in the absence of any commercial or financial relationships that could be construed as a potential conflict of interest.

The reviewer MZ declared a shared affiliation, with one of the authors LS to the handling editor at the time of the review

## Publisher’s Note

All claims expressed in this article are solely those of the authors and do not necessarily represent those of their affiliated organizations, or those of the publisher, the editors and the reviewers. Any product that may be evaluated in this article, or claim that may be made by its manufacturer, is not guaranteed or endorsed by the publisher.
