# Supplementary material for: Extra-Neural Metastases From Primary Intracranial Ependymomas: A Systematic Review
Source: Front Oncol. 2022 Apr 27;12:831016. doi: 10.3389/fonc.2022.831016 (PMC9093681; doi:10.3389/fonc.2022.831016)
Supplement: Supplementary file 1 [file Table_1.docx]

**Supplementary File 1.** Risk of bias assessments for included studies.

| **Joanna Briggs Institute Checklist for Case Reports – Criteria** |
| --- |
| 1. Were patient’s demographic characteristics clearly described? |
| 2. Was the patient’s history clearly described and presented as a timeline? |
| 3. Was the current clinical condition of the patient on presentation clearly described? |
| 4. Were diagnostic tests or assessment methods and the results clearly described? |
| 5. Was the intervention(s) or treatment procedure(s) clearly described? |
| 6. Was the post-intervention clinical condition clearly described? |
| 7. Were adverse events (harms) or unanticipated events identified and described? |
| 8. Does the case report provide takeaway lessons? |
| **Responses Options**: Yes, No, Unclear, Not Applicable (NA) |
| **Quality Rating**: Poor 0 – 2; Fair 3 – 5; Good 6 – 8 |

| **Study (Case Report)** | **1** | **2** | **3** | **4** | **5** | **6** | **7** | **8** | **Rating** |
| --- | --- | --- | --- | --- | --- | --- | --- | --- | --- |
| Maass et al. – 1954 [9] | Yes | Yes | Yes | Yes | Yes | Yes | Yes | Yes | 8 – Good |
| Sherbaniuk et al. – 1956 [10] | Yes | Yes | Yes | Yes | Yes | Yes | Yes | Yes | 8 – Good |
| Breslich et al. – 1957 [21] | Yes | No | Yes | Yes | Yes | Yes | Yes | Yes | 7 – Good |
| Perry et al. – 1957 [32] | Yes | Yes | Yes | Yes | Yes | Yes | Yes | Yes | 8 – Good |
| Wen et al. – 1957 [43] | Yes | Yes | Yes | Yes | Yes | Yes | Yes | Yes | 8 – Good |
| Nigogosyan et al. – 1962 [48] | Yes | Yes | Yes | Yes | Yes | Yes | Yes | Yes | 8 – Good |
| Glasauer et al. – 1963 [49] | Yes | Yes | Yes | Yes | Yes | Yes | Yes | Yes | 8 – Good |
| MacMahon et al. – 1964 [50] | Yes | Yes | Yes | Yes | No | No | Yes | Yes | 6 – Good |
| Hesselvik et al. – 1965 [51] | Yes | Yes | Yes | Yes | Yes | Yes | Yes | Yes | 8 – Good |
| Wentworth et al. – 1966 [12] | Yes | Yes | Yes | Yes | Yes | Yes | Yes | Yes | 8 – Good |
| Robinson et al. – 1967 [13] | Yes | Yes | Yes | Yes | Yes | Yes | Yes | Yes | 8 – Good |
| Braun et al. – 1968 [14] | Yes | Yes | Yes | Yes | Yes | No | No | Yes | 6 – Good |
| Smith et al. – 1969 [15] | Yes | Yes | Yes | Yes | Yes | No | No | Yes | 6 – Good |
| Hojgaard et al. – 1970 [16] | Yes | Yes | Yes | Yes | Yes | No | No | Yes | 6 – Good |
| Tamura et al. – 1980 [18] | Yes | Yes | Yes | Yes | Yes | No | No | Yes | 6 – Good |
| Duffner et al. – 1981 [19] | Yes | Yes | Yes | Yes | Yes | Yes | Yes | Yes | 8 – Good |
| Panyathanya et al. – 1982 [20] | Yes | Yes | Yes | Yes | Yes | No | No | Yes | 6 – Good |
| Andoh et al. – 1984 [22] | Yes | No | Yes | Yes | Yes | Yes | Yes | Yes | 7 – Good |
| Ferracini et al. – 1984 [23] | Yes | Yes | Yes | Yes | Yes | Yes | Yes | Yes | 8 – Good |
| Wakabayashi et al. – 1986 [24] | Yes | Yes | Yes | Yes | Yes | No | No | Yes | 7 – Good |
| Liote et al. – 1988 [25] | Yes | Yes | Yes | Yes | Yes | Yes | Yes | Yes | 8 – Good |
| Itoh et al. – 1990 [26] | Yes | Yes | Yes | Yes | Yes | Yes | Yes | Yes | 8 – Good |
| Strunk et al. – 2003 [29] | Yes | Yes | Yes | Yes | Yes | Yes | Yes | Yes | 8 – Good |
| Kinoshita et al. – 2004 [30] | Yes | Yes | Yes | Yes | Yes | Yes | Yes | Yes | 8 – Good |
| Kumar et al. – 2007 [31] | Yes | Yes | Yes | Yes | Yes | No | Yes | Yes | 7 – Good |
| Donepudi et al. – 2009 [33] | Yes | Yes | Yes | Yes | Yes | Yes | Yes | Yes | 8 – Good |
| Hussain et al. – 2010 [34] | Yes | Yes | Yes | Yes | Yes | Yes | Yes | Yes | 8 – Good |
| Chao et al. – 2011 [35] | Yes | Yes | Yes | Yes | Yes | Yes | Yes | Yes | 8 – Good |
| Davis et al. – 2011 [36] | Yes | Yes | Yes | Yes | Yes | Yes | Yes | Yes | 8 – Good |
| Fischer et al. – 2013 [37] | Yes | Yes | Yes | Yes | Yes | Yes | Yes | Yes | 8 – Good |
| Alzahrani et al. – 2014 [38] | Yes | Yes | Yes | Yes | Yes | Yes | Yes | Yes | 8 – Good |
| Pachella et al. – 2015 [39] | Yes | Yes | Yes | Yes | Yes | Yes | Yes | Yes | 8 – Good |
| Tyzo et al. – 2015 [40] | Yes | Yes | Yes | Yes | Yes | Yes | Yes | Yes | 8 – Good |
| Kim et al. – 2017 [41] | Yes | Yes | Yes | Yes | Yes | Yes | Yes | Yes | 8 – Good |
| Marsecano et al. – 2017 [42] | Yes | Yes | Yes | Yes | Yes | Yes | Yes | Yes | 8 – Good |
| Umbach et al. – 2019 [44] | Yes | Yes | Yes | Yes | Yes | Yes | Yes | Yes | 8 – Good |
| Shunnan et al. – 2020 [45] | Yes | Yes | Yes | Yes | Yes | Yes | Yes | Yes | 8 – Good |
| St Jeor et al. – 2020 [46] | Yes | Yes | Yes | Yes | Yes | Yes | Yes | Yes | 8 – Good |

| **Joanna Briggs Institute Checklist for Case Series – Criteria** |
| --- |
| 1. Were there clear criteria for inclusion in the case series? |
| 2. Was the condition measured in a standard, reliable way for all participants included in the case series? |
| 3. Were valid methods used for identification of the condition for all participants included in the case series? |
| 4. Did the case series have consecutive inclusion of participants? |
| 5. Did the case series have complete inclusion of participants? |
| 6. Was there clear reporting of the demographics of the participants in the study? |
| 7. Was there clear reporting of clinical information of the participants? |
| 8. Were the outcomes or follow up results of cases clearly reported? |
| 9. Was there clear reporting of the presenting site(s)/clinic(s) demographic information? |
| 10. Was statistical analysis appropriate? |
| **Responses Options**: Yes, No, Unclear, Not Applicable (NA) |
| **Quality Rating**: Poor 0 – 3; Fair 4 – 7; Good 8 – 10 |

| **Study (Case Series)** | **1** | **2** | **3** | **4** | **5** | **6** | **7** | **8** | **9** | **10** | **Rating** |
| --- | --- | --- | --- | --- | --- | --- | --- | --- | --- | --- | --- |
| Perrin et al. – 1958 [47] | Yes | Yes | Yes | Yes | Yes | Yes | Yes | Yes | Yes | NA | 9 – Good |
| Fragoyannis et al. – 1966 [11] | Yes | Yes | Yes | Yes | Yes | Yes | Yes | Yes | No | NA | 8 – Good |
| Schuster et al. – 1976 [17] | Yes | Yes | Yes | Yes | Yes | Yes | Yes | Yes | Yes | NA | 9 – Good |
| Newton et al. – 1992 [27] | Yes | Yes | Yes | Yes | Yes | Yes | Yes | Yes | Yes | NA | 9 – Good |
| Fouladi et al. – 2003 [28] | Yes | Yes | Yes | Yes | Yes | Yes | Yes | Yes | No | NA | 8 - Good |
